# Dry Powder Inhalers: A Focus on Advancements in Novel Drug Delivery Systems

**DOI:** 10.1155/2016/8290963

**Published:** 2016-10-27

**Authors:** Piyush Mehta

**Affiliations:** Dry Powder Inhaler Lab, Respiratory Formulations, Cipla R & D, LBS Road, Vikhroli (W), Mumbai, Maharashtra 400079, India

## Abstract

Administration of drug molecules by inhalation route for treatment of respiratory diseases has the ability to deliver drugs, hormones, nucleic acids, steroids, proteins, and peptides, particularly to the site of action, improving the efficacy of the treatment and consequently lessening adverse effects of the treatment. Numerous inhalation delivery systems have been developed and studied to treat respiratory diseases such as asthma, COPD, and other pulmonary infections. The progress of disciplines such as biomaterials science, nanotechnology, particle engineering, molecular biology, and cell biology permits further improvement of the treatment capability. The present review analyzes modern therapeutic approaches of inhaled drugs with special emphasis on novel drug delivery system for treatment of various respiratory diseases.

## 1. Introduction

Development of inhalation technology for drug delivery has contributed immensely in treating various intrapulmonary and extrapulmonary diseases. This is supported by the lungs' unique geometry such as large surface area, thin alveolar epithelial lining, high vascularization, and avoidance of first-pass metabolism. Numerous inhalation delivery systems have been developed and studied to treat lung diseases such as asthma, COPD, and other pulmonary infections. Among them, three approaches, that is, nebulizers, pressurized metered-dose inhalers (pMDIs), and dry powder inhalers (DPI), are extensively scrutinized for the treatment of several lung diseases and pathological conditions. Each type of delivery has unique strengths and weaknesses in view of the class of drugs that can be applied, type of formulation that can be utilized, and the sum of respirable dose that can be delivered from devices. Utilization of nebulizers requires bulky compressors or a source of compressed air, while pMDIs have limitations such as sedimentation, crystal growth, and selection of appropriate propellant and they emit dosages at high velocity, which build deposition in the oropharynx most commonly where they are swallowed and enhance the risk of systemic absorption [1]. DPIs were introduced to defeat some of the weaknesses associated with nebulizers and pMDIs [2].

DPIs provide better physicochemical stability and deep lungs deposition using the patient's respiration. Additionally, they do not require cold chain storage or reconstitution of powders into solutions for nebulization [1, 2]. Lactose monohydrate is the most commonly used excipient (i.e., carrier) in DPI formulations. Mixtures of the lactose with drug are commonly described as ordered or interactive mixtures, which are simpler to handle during the manufacturing than micronized drug alone. The drug particles should loosely adhere to the lactose (carrier) particles and, during inhalation in the turbulent air stream which is produced, the drug particles detach from the lactose (carrier) particles and are made accessible for deposition into the lungs. The larger carrier particles normally impact the oropharyngeal region. The carrier also offers mass to the DPIs, which enhances the handling, dispensing, and actuation of the micronized drug, which is of actual significance for low dose DPIs such as steroids (typical dose per actuation: 50 *μ*g to 500 *μ*g) [3, 4]. In order to confirm effective delivery of drug, it is crucial that adhesive forces between the drug and carrier are not so strong that separation from the carrier is prohibited. The equilibrium between adhesive and cohesive forces should be in tune to confirm sufficient adhesion between drug and carrier so as to provide a stable formulation (homogeneous blend with good content uniformity) but with adequate detachment of drug from carrier on inhalation. It has been recognized that the efficiency of a DPI formulation is greatly dependent on the particle size distribution, fine-lactose content, lactose source, the inhalation flow rate, and dispersion capacity of the respective DPI device [3].

With the growth of disciplines such as biomaterials and materials science, nanotechnology, particle engineering, molecular biology, and cell biology have gotten rapid development in field of novel drug delivery systems (NDDS). These NDDS in the field of western medicine have been extensively studied and applied clinically. The development of NDDS based DPIs has the potential to overcome issues associated with carrier as a critical component of the formulation. Moreover, they are expected to accomplish various key requisites like aerosolization properties of the formulation, physicochemical characteristics of powder, stability, biocompatibility, and biodegradation without any adverse effect. Conservative delivery systems are unable to meet these demands. Developing various novel drug delivery systems, for instance, liposomes, solid lipid nanoparticles, polymeric nanoparticles, nanoaggregates, nanocomposites, polymeric microparticles, and microspheres, has offered a number of advantages for traditional dry powder inhaler, including physical and chemical stability, flow properties, dispersion, tissue distribution, and bioavailability. Several potential DPIs have been successfully delivered using NDDS approach. Therefore, in this article, an attempt has been made to touch upon different aspects related to the development of novel drug delivery systems including their impact on drug dispersion and disposition and other physicochemical properties are discussed in detail. This review offers a perspective on current reported studies to modify pulmonary deliveries for its better functioning.

## 2. Novel Drug Delivery System for DPI

### 2.1. Lipid Vesicles

#### 2.1.1. Liposomes

Liposomes are typically self-enclosed small spherical arrangement composed of a single bilayer lipid membrane (unilamellar liposomes) or several bilayer lipid membranes (multilamellar liposomes) with a size range of 50 to 1000 nm. They have various functions ranging from drug delivery to food carrier [5]. They help improve the solubility, bioavailability, in vitro and in vivo stability, targeted delivery, and sustained release and, moreover, have ability to enhance the intracellular uptake of drug components and biodistribution pattern with protecting the encapsulated agent from the destructive action of the external environment [6]. Literature showed that numerous inhaled liposomal products are in various stages of clinical trials [7]. Site-specificity, biodegradability, biocompatibility, good safety profile, and potential to enclose both hydrophobic and hydrophilic particles are the prime reasons to choose liposomes in pharmaceutical industries [8].

Liposomes are widely scrutinized as vehicle for the dry powder inhalation delivery of various drugs for treatment of several diseases and pathological conditions. They can be easily aerosolized and provide prolonged retention of carriers and drugs in the respiratory track [22]. Liposomes seem mostly suitable for delivery of drugs to the lungs as they can be prepared from substances endogenous to the lung as elements of lung surfactant. Lung surfactant is a complex mixture, of which about 85% is phospholipid, generally dipalmitoyl phosphatidylcholine (DPPC), with phosphatidylglycerol as the next most widespread phospholipid. Surfactant is also composed of cholesterol and two groups of nonserum proteins which are believed to be important in the adsorption, spreading, and reutilization of surfactant. The mechanisms for the clearance and reutilization of lung surfactant are likely to be of major importance in determining the fate of liposomes deposited in the alveoli. The rate and extent of pulmonary uptake of liposomes are a function of their composition; significantly faster rates occur when liposomes contain phosphatidylglycerol [23]. In most cases, no considerable undesirable side effects were observed after the application of neutral or slightly negatively charged liposomes, though cationic liposomes were found to be toxic to human cells and potentially can introduce genetic aberrations [24]. Besides, adverse side effects of cationic liposomes considerably increased with an increase of positive charge of the particles. Still, because cationic liposomes normally are used for the formation of almost neutral complexes with negatively charged nucleic acids, such modification of cationic carriers usually prevents adverse effects on the cells [24]. Various investigations on liposomal formulations of inhaled drugs are listed in Table 1.

#### 2.1.2. Proliposomes

Proliposomes are described as dry, free-flowing microparticles with a dispersed system which can easily form a liposomal vesicle when in contact with water or biological fluids. Simply, powdered drugs are prepared by the adsorption of phospholipids and drug moiety onto the microporous matrix of carrier particles [37]. Compared with conventional liposomes, proliposomes display more advantages in stability, drug release, and solubility [21]. Therefore, proliposomes would be a potential medium to enhance oral absorption of hydrophobic drugs. The primary mechanism allowing proliposomes to improve oral absorption might be partially explained by the presence of bile salts, which can interact with phospholipids in the gastrointestinal tract to construct mixed micelles for medium to augment the solubility of hydrophobic drugs [38]. Various investigations on proliposomal formulations of inhaled drugs are listed in Table 1.

#### 2.1.3. Lipospheres

Lipospheres are also typically self-enclosed small spherical arrangement composed of water dispersible solid microparticles of particle size between 0.2 and 100 *μ*m in diameter and a solid hydrophobic fat core stabilized by one monolayer of phospholipid molecules embedded in their surface. Due to their solid lipid matrix, controlled release from these carriers is achievable which is essential to provide the drug over a prolonged period of time with improved bioavailability and physicochemical stability [42].

Cyclosporine A (CsA) lipospheres based DPI was formulated using dipalmitoylphosphatidylcholine (DPPC) and dipalmitoylphosphatidylglycerol (DPPG) (75 : 25) phospholipids by spray drying method. Aerosol dispersion pattern was determined using a next generation impactor (NGI) with a HandiHaler device at 60 L/min using #3 HPMC capsules. The percentage of fine particle fraction (FPF), mass median aerodynamic diameter (MMAD), and geometric standard deviation (GSD) was found to be 52.99 ± 4.12%, 2.79 ± 0.47 *μ*m, and 1.85 ± 0.05, respectively. In summary, compared to drug alone prepared co-SD CsA, DPPC/DPPG lipospheres displayed better physicochemical properties and aerosol dispersion with very low residual water content [63]. Singh et al. investigated the rifampicin loaded phospholipid lipospheres containing sulfphobutyl ether, *β*-cyclodextrin, and Vit. C formulated using spray drying technique. Prepared amorphous lipospheres showed spherical morphology with the size range of 1–5 *μ*m. Aerosol dispersion pattern was concluded using Andersen cascade impactor (ACI) with device operating at 28.3 L/min using #3 HPMC capsules. The FPF and emitted dose (ED) were found to be 52.99 ± 4.12% and 85.40 ± 1.04%, respectively. Moreover, prepared lipospheres exhibited approximately ~2-fold improvement in antimycobacterial activity over MTB H_37_Rv strain compared to drug alone. Improvement in antimycobacterial activity may be attributed to the cyclodextrin-cholesterol matrix structure [64].

#### 2.1.4. Lipid-Coated Particles

Liposomes, proliposomes, and lipospheres have been the platform of choice for pulmonary drug delivery applications over the past decade. However, extensive investigation has revealed their restrictions as drug delivery carriers. They have limitations. For instance, phospholipid may sometimes undergo oxidation and hydrolysis like reaction, short half-life, and poor mechanical stability due to leakage and fusion of the formulation [70]. To overcome these limitations, a hybrid class of lipid-coated particles has received good attention in recent years. Lipid coating offers better stability and higher encapsulation efficiency than liposomes. The existence of a low lipid coating level permits the preparation of powders with few excipients, thereby delivering more drug to the lungs. Additionally, the hydrophobic nature of neutral lipids (cholesterol) decreases the absorption of the ubiquitous vapor, leading to lessening the aggregation and the adhesion of particles [71].

Tobramycin lipid-coated particles were prepared using mixtures of cholesterol and phospholipids (75 : 25) by spray drying technique. Developed lipid-coated particles showed small, smooth surface properties and good flowability with a mean size of about 1-2 mm. Aerosol dispersion pattern was determined using a multistage liquid impinger (MSLI) with a Cyclohaler® device at 100 L/min for 2.4 s using #3 HPMC capsules containing 15 mg of tobramycin lipid-coated particles. The FPF of prepared lipid-coated particles was found to be 68% with approximately ~1.5-fold improvement in FPF compared to uncoated micronized tobramycin particles. Developed lipid-coated tobramycin DPI formulations required very low amount of excipient and produce better lung deposition. These formulations are mostly useful for drugs that are active at relatively high doses, for example, antibiotics, since they allow the delivery of a high concentration of antibiotic directly to the site of action while minimizing systemic exposure [71].

### 2.2. Nanoparticulate System

Nanoparticles are solid, colloidal particles comprising macromolecular materials that differ in size from 10 nm to 1000 nm in which the drug moiety is dissolved, encapsulated, and entrapped and/or to which the drug moiety is adsorbed, embedded, or attached, which can be administered in fluid form with a liquid carrier. A wide variety of delivery systems includes nanoparticles, polymeric nanoparticles, polymeric micelles, solid lipid nanoparticles (SLNs), nanostructured lipid carriers (NLCs), nanocapsules, nanospheres, nanoemulsion, and many more. They have the capacity to modulate both the pharmacokinetic and pharmacodynamic properties of drugs, thereby improving their therapeutic index. Nanoparticles help modify the biodistribution, in vivo stability, bioavailability, and penetration through biological barriers. Moreover, they also provide controlled and targeted drug release [81–83].

Inhalable nanoparticles are advantageous for the objective of improving the solubility, dissolution profiles, and pharmacokinetic profile and reducing premature mucociliary clearance of hydrophobic drugs [84]. The size, shape, and composition of nanoparticles are key features which determine their percentage retention and targeting properties [22]. Inhaled nanoparticles could be easily exhaled during administration due to their small diameter (≪1 *μ*m) and low inertia. Nanonization often conveys an enormous increase in Gibb's free energy due to the increase in surface area. Moreover, these particles have tendency to grow due to Ostwald ripening and can suffer from uninhibited agglomeration [85–88]. This issue has been resolved by transforming nanoparticles into inhalable microaggregates via numerous techniques like spray drying and spray freeze drying or with leucine, mannitol, and PVA as matrix components [89, 90]. Spray freeze dried microaggregates exhibited superior aerosolization performance compared to those produced by spray drying and this was credited to the porous structure and low density of the particles [91–93]. Various investigations on nanoparticulate formulations of inhaled drugs are listed in Table 2.

#### 2.2.1. Solid Lipid Nanoparticles and Nanostructured Lipid Carriers

SLNs are the first generation of lipid nanoparticles. They are colloidal carriers made up of physiologically tolerated lipids components that remain solid at body and room temperature with mean diameter of 40 to 1000 nm [94]. Generally, SLNs are made up of 0.1–30% (w/w) lipid dispersed in an aqueous solution of 0.5–5% (w/w) surfactant as stabilizing agent. SLNs provide different advantage like physicochemical stability, protection of the incorporated drug from degradation, prolonged/sustained release profile with pseudo-zero-order kinetics, and low cytotoxicity, if well tolerated excipients are used [95, 96]. SLNs have disadvantages like low drug loading capacity and drug expulsion during storage. Drug expulsion takes place when the low ordered lipid modification of the particle matrix after manufacturing switches to the highly ordered *β*-modification during storage. The *β*-modification is characterized by a perfect crystal lattice with few imperfections and hence little room for drug accommodation. To defeat these SLNs related issues, the second generation of lipid nanoparticles, nanostructured lipid carriers, was developed [97]. NLCs are a delivery system in which partial-crystallized lipid particles with mean particle size ≤100 nm are dispersed in an aqueous phase containing emulsifier(s). NLCs have a solid lipid matrix at room and body temperature which is composed of a mixture of a solid lipid and oil, preferable in a ratio of 70 : 30 up to a ratio of 99.9 : 0.1. Through mixing different kinds of lipids, a less ordered lipid matrix with high drug loading capacity for drugs is achieved [95].

Moreover, due to their controlled size, nanoparticles can be easily aerosolized into droplets with aerodynamically suitable properties, which permits adequate deep lung deposition of a drug. Furthermore, particle adhesion, accumulation, and retention in the pulmonary region as well as controlled release properties of SLNs and NLCs may lead to improved and sustained therapeutic outcomes, resulting in a longer dosing interval and better patient compliance [95, 98]. Considering these unique functionalities, researchers have developed several SLNs and NLCs based DPI formulations to enhance the therapeutic efficacy of inhaled drug molecules (Table 3).

### 2.3. Microparticulate System

The expression “microparticle” in drug delivery is usually designated to a particle with one or several micrometers in dimension. Based upon the method of preparation, the drug is entrapped, encapsulated, or simply dissolved to the microparticle template. Microparticle matrix contains many materials like polymers, ceramics, metals, and glass. This system is believed and accepted as a steady means to deliver the drugs to the target site with specificity [57]. Various delivery approaches involve microparticles, microspheres, solid lipid microparticles, polymeric microparticles [58, 99], surface coated microparticles [61], and so forth. In clinical practices, microparticles appreciably influence retention in the lungs and targeting properties of therapeutic agent, apart from the fact that active targeting microparticles also offer numerous advantages like masking, facilitation of handling, controlled dissolution profile, and protection [100]. Various investigations on microparticulate formulations of inhaled drugs are listed in Table 4.

#### 2.3.1. Microsphere

Microspheres are microparticles of spherical shape without membrane or any distinct outer layer. They are small solid spherical particles made up of various natural and synthetic materials with mean diameter of 1 to 1000 *μ*m. Microspheres may release drug at controlled rates for long periods of time. Microspheres are ideal vehicles for many controlled delivery applications due to their ability to encapsulate a variety of drugs, biocompatibility, high bioavailability, and prolonged/sustained drug release characteristics. Such techniques also offer various benefits over conventional methods of drug delivery, including protection of fragile drugs, tailoring of drug release rates, and increased patient comfort and compliance. Polymethyl methacrylate, acrolein, glycidyl methacrylate, lactides, poly(alkyl cyanoacrylate), polyanhydrides, poly(D, L-lactic-co-glycolic acid) and chitosan, poly(acryl) dextran, poly(acryl) starch, carrageenan, and gelatin are the commonly used polymeric agents for microspheres synthesis [101]. Microspheres based DPI formulations are listed in Table 5.

#### 2.3.2. Mucoadhesive Microparticles

Mucoadhesion can be defined as the interaction of molecules with the mucous layer (biological tissue) in order to adhere to each other. Mucoadhesion techniques have received a good attention, in drug delivery system, due to their two prime requisites. First, they increase mean residence time of therapeutic agent. Second, they uphold a high concentration gradient of drug molecule across the epithelium. Mucoadhesion occurs via several mechanisms such as chain interlocking, diffusion, and chemical reaction (hydrogen bonding) [102]. These mucoadhesive agents are usually high molecular weight polymers which can interact with the mucin layer of the respiratory epithelium due to hydrogen bonding and electrostatic, hydrophobic, or van der Waals interactions. Chitosan, carbomer, poly(lactic acid), poly(D,L-lactic-co-glycolic acid), hydroxypropyl cellulose, and sodium carboxy methylcellulose are the commonly used mucoadhesive agents for inhalation drug delivery. The incorporation of therapeutic agents into these polymeric matrices regulates the drug pharmacokinetics by improving lung mean residence time. Even though the idea of mucoadhesion at the upper respiratory tract has been broadly studied, application to the lower respiratory tract has not been explored [103].

M. Mishra and B. Mishra formulated the doxycycline hyclate mucoadhesive microparticles using sodium carboxy methyl cellulose as mucoadhesive agents by spray drying technique. Developed microparticles showed moderately dimpled but spherical shape (1–5 *μ*m) with 56.27% yield, 83.74 ± 2.31% encapsulation efficiency, and 4.16 ± 0.52% water content. Aerosol dispersion was examined using ACI with monodose inhaler device at flow rate of 60 L/min for 4 sec using #2 gelatin capsules. ACI analysis suggested good dispersion and deposition with the FPF, ED, and MMAD of 55.5 ± 4.2%, 80.2 ± 0.4, and 4.56 ± 0.10, respectively. Drug release from these mucoadhesive microparticles showed biphasic Fickian type of diffusion. Moreover, prepared microparticles exhibited good output in MTT assay on H1299 mammalian alveolar cells. Doxycycline hyclate containing sodium carboxy methyl cellulose mucoadhesive microparticles would be helpful dosage form for clinical treatment of respiratory infections with minimal adverse side effects [103]. Mucoadhesive beclomethasone dipropionate (BDP) microspheres for powder inhalation were formulated using hydroxypropyl cellulose (HPC) mucoadhesive polymer (1 : 4) by spray drying technique. This resulted in amorphous (aBDP/HPC) and crystalline (cBDP/HPC) microspheres with mean particle size of 2.5–2.9 *μ*m. BDP's aqueous solubility was improved 25 times for aBDP/HPC, compared to cBDP/HPC. In antigen-induced asthmatic Guinea pigs, aBDP/HPC showed quick BDP absorption from the lungs (≥95% absorption for 180 min) with a greater metabolite (B17MP) formation, compared to cBDP/HPC, mainly due to the improved dissolution of aBDP/HPC. In contrast, 86.0% of BDP remained at 180 min following cBDP/HPC administration, indicating the prolonged retention of BDP in the lungs by virtue of poor dissolution (release) and delayed mucociliary clearance. cBDP (1.37 mg/kg) alone considerably inhibited eosinophil infiltration into the lungs of antigen sensitized and challenged Guinea pigs for only 1–6 h and at much lower drug dosage of cBDP/HPC (0.25 mg/kg) was capable of maintaining such inhibitory effects for 24 h following administration. The prolonged lung retention of BDP by the use of the cBDP/HPC microspheres was credited to prolonging its pharmacological duration without requiring increased drug dosage [104].

### 2.4. Solid Dispersions

Solid dispersions are one of the known approaches to improve drug solubility and release profile of poorly water-soluble drugs. It is a molecular mixture of drug in hydrophilic polymer wherein the drug physicochemical characteristics are regulated by the polymer properties [105]. Here, the drug dissolution profile may be enhanced by reducing the drug particle size to nearly a molecular level and by transforming the drug crystalline state to create a complete or partially amorphous state, both of which may improve drug solubility and bioavailability [106]. Commonly used polymers in formulation of solid dispersions are cyclodextrin, hydroxy propyl methylcellulose, hydroxy propyl cellulose, ethyl cellulose, silica, povidone, starch, and polyethylene glycols [107]. Solid dispersions act as a flexible platform for novel drug delivery systems.

Itraconazole (ITZ) SD based DPI was formulated using polymeric surfactant, that is, TPGS and hydrophilic agent, and mannitol by spray drying method. Prepared ITZ-SD-DPI compared with ITZ-SD-DPI without TPGS and bulk ITZ. ITZ-SD produced an improved dissolution rate and greater saturation solubility than bulk ITZ. The use of a polymeric surfactant (TPGS) was helpful in terms of solubility and dissolution profile but it also reduced FPF (47 ± 2% to 37.2 ± 0.4%). Additionally, device and capsule retention of ITZ-SD-DPI was significantly reduced when TPSG was used into the formulations. This may be due to lubricant effect of TPGS which improved device and capsule emptying by reducing the surface interaction and greater particle cohesion. Mannitol solid dispersions with optimized quantity polymeric surfactant may provide an effective platform for poorly soluble active ingredients DPI [108]. Cyclosporine A (CsA) amorphous solid dispersion was prepared by using wet-milling technique (WM/CsA) and jet milled with coarse lactose (Respitose SV003) to obtain desired aerodynamic particle size. WM/CsA-DPI exhibits the better dissolution profile as compared to pure CsA. Aerosol dispersion was examined using ACI with Jethaler device operating at a flow rate of 28.3 L/min. ACI analysis recommended good dispersion and deposition in the respiratory organs with the FPF and ED of 54 and 96%, respectively. Moreover, intratracheal administration of WM/CsA (100 *μ*g CsA) in experimental animals led to 85 and 71% reduction of granulocyte recruitment in lung tissues and bronchoalveolar lavage fluids. In comparison to a commercially available CsA oral formulation, WM/CsA showed 10^2^-fold reduction in AUC and Cmax values of plasma CsA at toxic concentration (10 mg/kg). WM/CsA-DPI would be effective dosage form for clinical treatment of airway inflammations with minimal systemic side effects [109]. Δ^9^-Tetrahydrocannabinol solid dispersion was formulated using inulin in a mixture of tertiary butanol and water by spray freeze drying technique. Prepared formulation contains highly porous particles with specific surface area of 90 m^2^/g. In vitro deposition of the powder formulations was determined with MSLI at flow rate of 60 L/min for 3 sec. FPF was found to be 50% for prepared formulation [110].

### 2.5. Thin Film Freezing

The particle engineering process, thin film freezing (TFF), has ability to produce low density pharmaceutical powders with a porous, matrix structure. In TFH, the particle morphology can be controlled by influencing the fluid dynamics and heat transfer characteristics upon spreading and freezing of liquid droplets on solid surfaces. When stabilizing excipients with high glass transition temperatures, for instance, PVP or HPMC, are incorporated in the formulation, the pharmaceutical powders can be turned into amorphous ones. These low density TFF developed powders have also been shown to be highly respirable when aerosolized with a marketed inhaler [111, 112].

Microstructured crystalline voriconazole (VRC) and nanostructured amorphous TFF-VRC particles [PVP K25 (1 : 3)] were formulated using thin film freezing technique. The prepared microstructured crystalline VRC and nanostructured amorphous VRC particles showed particle size of 0.45 *μ*m and 0.05 *μ*m, respectively. Powder dispersion was examined using NGI with single dose capsule based HandiHaler device operating at a flow rate of 60 L/min. NGI analysis showed better dispersion and deposition pattern for microstructured crystalline VRC (% FPF 37.80) compared to nanostructured amorphous VRC (% FPF: 32.40) particles. Microstructured crystalline VRC particles also showed approximately ~2-fold improvement in % FPF in comparison to micronized VRC with InhaLac® 70 (2% w/w). Moreover, in single dose 24 h pharmacokinetic studies (10 mg/kg) microstructured crystalline VRC particles showed 2.06-fold improvement in AUC_(0–24 h)_ of plasma compared to nanostructured amorphous VRC particles [111]. Similarly, an amorphous dry powder of tacrolimus (TAC) was prepared using thin film freezing technique. Aerosol dispersion of TFF-processed powder was performed using Miat® monodose inhaler device. TFF-processed TAC dry powder showed FPF and MMAD of 83.30% and 2.26 *μ*m respectively. Although being of relatively large geometric size, the TFF-processed dry powder was capable of attaining deep lung due to its low density, highly porous, and brittle characteristics compared to a crystalline micronized TAC powder produced by milling technique. Additionally, in single dose 24 h pharmacokinetic study, TFF-processed powder showed better pulmonary bioavailability with prolonged retention time compared to a crystalline micronized TAC [112].

## 3. Protein, Peptides, and Vaccine Delivery

Numerous therapeutic proteins and peptides have been used for long time in routine clinical practice. These include insulin, human growth hormone (hGH), calcitonin, and parathyroid for treatment of diabetes, hormone deficiency, and osteoporosis, respectively. Due to recent advance in biochemical sciences and in the knowing of the physiopathology of many disorders, therapeutic biomolecules are likely to become of increasing significance. Pulmonary delivery of these proteins and peptides could compose an alternative to systemic delivery. In fact, direct administration to the lungs for local action allows high doses of protein and peptides to be distributed while preventing systemic adverse effects [113]. Another budding use of inhaled proteins and peptides is vaccination, as most antigens are macromolecules. Parenteral drug delivery is usually helpful in preventing systemic infections, but it fails to avoid the early interaction of pathogen and host and continual colonization at the mucosal surface. Mucosal immunization is the local and direct release of antigen to a mucosal surface to stimulate specific immunity versus an attacking pathogen. It has the ability to eradicate many of the difficulties linked with parenteral vaccinations [114]. For example, influenza vaccines have been shown in various animal models to induce appreciably superior mucosal antibody titres when served directly to the pulmonary region, equated with s.c. or i.m. injection [115, 116]. Additionally, pulmonary release can be utilized to offer systemic action of some proteins and peptides. Due to the high surface area of the lungs, the thin layer of the alveolar epithelium, and the high degree of lung vascularization, pulmonary delivery can present fast systemic absorption while ignoring the degradation mechanisms of the hepatic first-pass metabolism and gastrointestinal tract. Apart from this, pulmonary delivery offers advantages over injection in terms of both formulation and delivery technology, that is, patients' acceptance (needle-free delivery) and ease of administration [117].

However, delivery of proteins and peptides to the lungs presents some challenges. A prime issue with inhaled drugs is the necessity to provide the drug as microparticles (1–5 *μ*m) to reach the lungs. Secondary question, which is specific to inhaled proteins and peptides, is that they can suffer physicochemical degradation. Physicochemical degradation can also happen during handing out of the proteins and peptides due to diverse stresses, such as exposure to extreme pH and temperatures. These physicochemical degradations can lead to loss of therapeutic activity. Another concern with these biomolecules is that they are prone to in vivo biological degradation due to the presence of enzymes (i.e., proteases) in the lungs and assorted clearance in the pulmonary area and the blood [95, 118]. To circumvent these issues, different strategies have been tried and tested for pulmonary applied proteins and peptides. An overview of these strategies along with their key achievements is denoted in Table 6.

## 4. Miscellaneous

Theophylline (THF) cocrystals DPI was prepared using various conformers like urea, saccharin, and nicotinamide by spray drying method. Aerosol dispersion was examined using NGI with Aerolizer inhaler device operating at a flow rate of 60 L/min for 6 sec. THF nicotinamide cocrystals show better aerosolization performance in comparison to other cocrystals. Micromeritic properties of THF cocrystals play important role in terms of the aerosol performance [119]. Voriconazole loaded large porous particles (VLPP) were prepared from polylactide polymer for treatment of invasive pulmonary aspergillosis. The optimized VLPP exhibited 4.85 ± 0.39% loading efficiency and 0.17 ± 0.02 porosity. Aerosol performance was examined using ACI. MMAD and FPF of optimized batch were found to be 2.85 ± 0.38 *μ*m and 27.3 ± 2.7%, respectively. VLPP was shown to sustain the release of voriconazole for over 7 days with prolonging the residence time of VRZ at the site of action. Moreover, VLPP does not show any cytotoxic and inflammatory (IL-8) effect over A549 cells [120]. Methotrexate microspheres were fabricated using porous poly-L-lactide (MTX-PLLA-PMs) by emulsion combined precipitation of compressed CO_2_ antisolvent method. Prepared MTX-PLLA-PMs demonstrated a porous and uneven morphology with 81.6% of encapsulation efficiency. Additionally, obtained microspheres exhibited 79–85% porosity, 0.4 g cm^−3^ density, 10–25 *μ*m geometric mean diameter, and FPF of 58–62%. Moreover, in in vitro antitumor assay MTX-PLLA-PMs possessed a sustained and better inhibitory activity compared with the neat MTX [121]. Inhalable dry powder composed of crystalline rifapentine and amorphous verapamil in presence of l-leucine was formed by spray drying method. When the dry powder was dispersed by an Osmohaler®, the total FPF of rifapentine and verapamil was 71.5 ± 2.0% and 77.4 ± 1.1%, respectively. Notably, the combination resulted in increased killing of* M. tuberculosis* H37Ra with acceptable IC_50_ values (62.5 *μ*g/mL) on both lung alveolar basal epithelial (A549) and human monocytic (THP-1) cell lines. Furthermore, the dry powder was stable after three-month period in 0% RH at 20 ± 3°C [122].

## 5. Challenges and Future Perspectives

**Figure 1:**
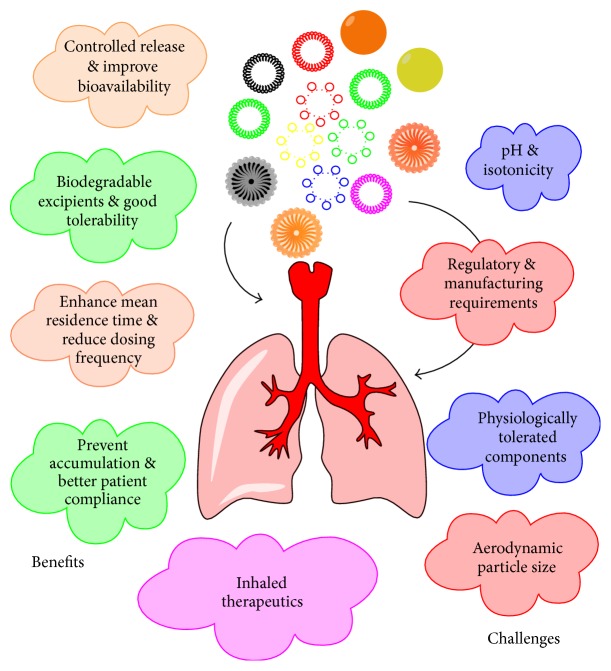
Advantages and challenges in novel respiratory drug deliveries.

The successful delivery of any therapeutic agent to the pulmonary region via a DPI depends on four mutually dependent parameters: the formulation, the metering system, the inhaler device, and the patient's understanding/training. This review describes in detail the novel formulation aspects of DPI and merely touched other parameters. But there is an evenly appealing detailed story to be told regarding the inhaler devices, metering system, and patient's understanding/training. Various advantages and challenges in novel respiratory application are given in Figure 1.

Cochleates, a new type of lipid based drug delivery system, are applicable for administration through various routes and can be formulated in powder form. The interior of a cochleate structure remains considerably intact irrespective of outer enzymatic conditions. They have been reported to improve the systemic bioavailability and safety with good level of patient compliance compared to liposomes. There is a need to develop cochleates based dry powder inhaler formulations [70]. Ligand conjugated delivery system can also be explored to enhance the efficacy of the dry powder inhaler. This targeted multifunctional approach not only enhances bioavailability but also helps to deliver therapeutic agents exclusively to the diseased cells in the lungs collectively with suppressors of their resistance to the therapy. Research efforts in this segment led to developed ligand conjugated based dry powder inhaler with better targeting approach.

The growth in NDDS has already boosted the use of DPI for a variety of therapeutic aliments. A lot of novel drug delivery systems have been employed to increase the efficacy of DPI. Still, novel polymer synthesis, use of functionalized carriers, evolution in particle engineering, and better knowledge of the structure function association have to be utilized to facilitate targeting of particles to predetermined lung cells. This may be tested by upholding of their structural integrity, biocompatibility, better stability in pulmonary fluids and enzymes, targeting ability, ease and safety of production, and regulatory requirements. For better localized targeting, ligand based drug delivery seems to have lot of prospect to reach the clinic in the near future. Ethosomes, cubosomes, and other vesicles as carrier systems are better alternative to extend the potential of DPI. Deposition efficiency, systemic toxicity, unfavorable PK/PD profile, and rapid lung clearance like critical issues can be defeated by improving the encapsulation efficiency and use of long circulating carriers. As detailed herein, there are a number of promising novel drug delivery options presently in the developmental pipeline, and if found to be safe, they will be valuable therapeutic alternative for clinicians to improve the end result and survival rates of patients with difficult-to-treat pulmonary infections.

## Figures and Tables

**Table 1:** Liposomes, nanoliposomes, and proliposomes inhaled formulation along with the key findings.

Drug	Method of preparation	Major ingredient	Assembly, device, capsules, and flow rate (L/min)	Percentage of FPF	Reference
*Liposomes*					
Amikacin sulfate	RPE followed by LP	HSPC, CS, and stearylamine	TSI, Rotahaler®, #2, and 60	29.2 ± 2.10	[9]
Budesonide	TFH followed by LP and BM	Egg phosphatidylcholine, CS, and *α*-tocopherol	TSI, Rotahaler, #2, and 60	20.69 ± 1.50	[10]
Dapsone	TFE followed by SD	DPPC, CS, and hydrolyzed gelatin	ACI, #2 HPMC, and 28.3	75.6 ± 1.60	[11]
Gemcitabine hydrochloride	SE followed by LP	HSPC, DSPG, mPEG_2000_-DSPE, and trehalose	ACI, #3 gelatin, and 60	56.12 ± 4.38	[12]
Ketotifen fumarate	TFH followed by FD	Saturated egg phosphatidylcholine, CS, and sucrose	TSI, #2, and 60 for 5 sec	21.59 ± 1.53	[13]
N-Acetylcysteine	RPE followed by SD	Phospholipid and CS	ACI, HandiHaler®, #3 gelatin, and 28.3 for 8.5 sec	35.34 ± 3.86	[14]
Recombinant secretory leukocyte protease inhibitor (rSLPI)	RE followed by LP and JM	1,2-Dioleoyl-sn-glycero-3-[phospho-L-serine] and CS	TSI, Spinhaler®, #2 gelatin, and 60	59.5 ± 5.40	[15]
Rifampicin	TFE, coated with chitosan and carrageenan, followed by SD	SPC, HSPC, and CS	NGI, Turbospin®, #2 gelatin, and 70	51.1 ± 2.30	[16]
Salbutamol sulfate	Vesicular phospholipid gel technique followed by LP and BM	SPC	TSI, Spinhaler, #2, and 60	41.51 ± 2.22	[17]

*Nanoliposomes*					
Amiloride hydrochloride	TFH and then passed through HPH, followed by SD	HSPC, CS, and mannitol	ACI, Rotahaler, #2 gelatin, and 28.3	67.6 ± 0.60	[18]
Tacrolimus	TFE and then passed through HPH, followed by SD	HPC and CS	ACI, #2 HPMC, and 28.3	71.1 ± 2.5	[19]

*Proliposomes*					
Isoniazid	SD	L-*α*-soybean phosphatidylcholine, CS, and 90% mannitol	ACI and 60	71 ± 2.0	[20]
Rifapentine	SD	HSPC and stearylamine	ACI, #2 gelatin, and 60 for 4 sec	92.5 ± 1.5	[21]

SD: spray drying; FD: freeze drying; LP: lyophilization; RE: rotary evaporation; TFH: thin film hydration; HPH: high pressure homogenizer; CS: cholesterol; SPC: soybean phosphatidylcholine; HPC: hydrogenated phosphatidylcholine; HSPC: hydrogenated soy phosphatidylcholine; DPPC: dipalmitoyl phosphatidylcholine.

**Table 2:** Example of nanoparticulate inhaled formulation along with the key findings.

Drug	Method of preparation	Major ingredient	Assembly, device, capsules, and flow rate (L/min)	Percentage of FPF	Reference
*Nanoparticles*					
Carvedilol	Antisolvent precipitation-ultrasonication followed by LP.	Pluronic F-127, sodium deoxycholate, and mannitol	ACI, Aerolizer®, #3, and 60 for 4 sec	81.90 ± 4.30	[25]
Calcium-disodium EDTA	Antisolvent precipitation followed by SD	Calcium-disodium EDTA and PVA	ACI, HPMC, and 60 for 5 Sec	67.35 ± 2.27	[26]
Salmon calcitonin	Ionic gelation method followed by SD	Sodium tripolyphosphate, chitosan, and Mannitol	ACI, glass inhaler device, and 60	63.50	[27]

*Nanoparticle agglomerates*					
Budesonide	Antisolvent precipitation-ultrasonication followed by LP	Lecithin, cetyl alcohol, L-leucine, PVP, and Span 85	ACI and 60	95.9 ± 1.5	[28]
Levofloxacin	Emulsification solvent-evaporation method followed by SFD	Polycaprolactone, mannitol, and leucine	NGI, powder entrainment tube, and 85	30.00	[29]
Fluticasone propionate and albuterol sulfate (2 : 1 w/w)	Antisolvent precipitation followed by precipitation and homogenization	Lecithin, cetyl alcohol, L-leucine, PVP-K90, and Pluronic F-127	ACI, #3 gelatin, and 28.3 for 4 Sec	Fluticasone propionate, 84 ± 7, and albuterol sulfate, 70 ± 3	[30]
Theophylline (nanorods agglomerates)	Antisolvent precipitation-ultrasonication followed by LP	Stearic acid	ACI, Aerolizer, and 60	81.9 ± 4.30	[31]

*Microparticulate/nanoparticulate systems*					
Azithromycin	co-SD	Methanol	NGI, HandiHaler, #3 HPMC, and 60 for 10 sec	53.45 ± 3.60	[32]
Paclitaxel	co-SD	DPPC and DPPG	NGI, HandiHaler, #3 HPMC, and 60 for 10 sec	86.7 ± 2.60	[33]
Paclitaxel	co-SD	DPPC and DPPE-PEG	NGI, HandiHaler, #3 HPMC, and 60 for 10 sec	77.9 ± 7.00	[34]
Tobramycin	co-SD	Methanol	NGI, HandiHaler, #3 HPMC, and 60 for 10 sec	28.48 ± 3.39	[32]
Tobramycin	Emulsion diffusion followed by co-SD	PLGA and PVA	MSLI, Turbospin, and gelatin	52 ± 1.00	[35]
Sodium cromoglicate	Liquid antisolvent precipitation followed by immediate SD	Deionized water	MSLI, monodose inhaler device, #3 HPMC, and 60	62.02 ± 2.12	[36]

SD: spray drying; co-SD: closed spray drying; FD: freeze drying; SFD: spray freeze drying; LP: lyophilization; PLGA: poly(lactic-co-glycolic acid); PVA: poly(vinyl alcohol); PVP: polyvinylpyrrolidone; DPPC: dipalmitoyl phosphatidylcholine; DPPG: dipalmitoylphosphatidylglycerol.

**Table 3:** SLN and NLC based DPI formulations.

Drug	Method of preparation	Major ingredient	Assembly, device, capsules, and flow rate (L/min)	Percentage of FPF	Reference
*Solid lipid nanoparticles*					
Thymopentin	Double emulsion technique followed by co-SD	Glyceryl monostearate and SPC (1 : 1, w/w), poloxamer 188, mannitol, and leucine	TSI, #3 HPMC, and 60 for 10 sec	51.07 ± 1.21	[39]

*Nanostructured lipid carriers*					
Montelukast	Melt emulsification-homogenization followed by LP	Precirol ATO5, Capryol-90, and mannitol	ACI, Rotahaler, #3 gelatin, and 60 for 4 sec	90.22 ± 2.60	[40]
Rosuvastatin	Melt-emulsification and ultrasonication followed by LP	Lauric acid, Capryol-90, mannitol, and L-leucine	ACI, Rotahaler, #3 gelatin, and 60	91.25 ± 3.57	[41]

co-SD: closed spray drying; LP: lyophilization; SPC: soybean phosphatidylcholine.

**Table 4:** Example of microparticulate inhaled formulation along with the key findings.

Drug	Method of preparation	Major ingredient	Assembly, device and capsules, flow rate (L/min)	Percentage of FPF	Reference
*Microparticles*					
Budesonide	SD	Lactohale 300 M : Pharmatose® 150 M (60 : 40)	ACI, Rotahaler, #3 HPMC, and 60 for 4 sec	34.50	[43]
Ciprofloxacin	co-SD	Mannitol	MSLI, Aerolizer, and 60 for 4 sec	43.5 ± 1.50	[44]
Ciclosporin	SFD	Tert-butyl alcohol and mannitol (5%)	ACI, Jethaler®, #2 HPMC, and 28.3 for 5 sec	36.4 ± 1.40	[45]
Dexamethasone	Vibrational SD	PCL and sodium deoxycholate	ACI, Aerolizer, #3 HPMC, and 28.3 for 8.5 sec	61.68 ± 1.28	[46]
Naringin	SD	Water-ethanol	TSI, Turbospin, #2 gelatin, and 60 for 5 sec	44.5 ± 1.54	[47]
Rifampicin	SD	Chitosan, ascorbic acid, and leucine	ACI, HPMC, and 60 for 10 sec	47.29	[48]
Rifampicin	SD	Dextran (Dex) and stearylamine grafted dextran	ACI and 60	77.51	[49]
Rifampicin	SD	Water : dichloromethane 80 : 20	ACI, Spinhaler, and 60 for 4 sec	58.00	[50]
Shuang-Huang-Lian corrugated particles	SD	D-Mannitol, lactose, and L-leucine	NGI, Cyclohaler®, #3 gelatin, and 60 for 4 sec	36.8 ± 1.90	[51]
Terbutaline sulfate	SD	L-leucine and low molecular wt. chitosan	MSLI, Spinhaler, #2 HPMC, and 60 for 2.5 sec	75.83 ± 2.59	[52]
Voriconazole	SD	Polylactide	ACI and RS01 dry powder inhaler	43.56 ± 0.13	[53]

*Solid lipid microparticles*					
Salbutamol sulfate	SD	DPPC, CS, and L-leucine	TSI, Cyclohaler, gelatin, and 60 for 4 sec	42.70	[54]
Quercetin	O/W emulsification via a phase inversion technique followed by SD	Tristearin and phosphatidylcholine	NGI, Aerolizer, #3 gelatin, and 60 for 4 sec	20.5 ± 3.30	[55]
Budesonide	O/W emulsification via a phase inversion technique followed by SD	Pluronic F-68 and Compritol 888	MSLI, Aerolizer, #3 HPMC, and 60 for 4 sec	21.1 ± 0.60	[56]

*Biopolymer based microparticles*					
Budesonide	Cation induced gelation method followed by LP	Sodium alginate, deacetylated chitosan, Pluronic F-68, and mannitol	ACI, Rotahaler, #3 HPMC, and 60 for 5 sec	60.09 ± 0.01	[57]
Budesonide	Cation induced gelation method followed by LP	Sodium alginate, deacetylated chitosan, Pluronic F-68, and mannitol	ACI, Rotahaler, #3 HPMC, and 60 for 5 sec	56.18 ± 0.01	[58]

*Miscellaneous microparticles*					
Beclomethasone dipropionate ultrafine particles	Microfluidic antisolvent precipitation followed by homogenization and SD	NA	MSLI, Aerolizer, #3 HPMC, and 60 for 4 sec	68.39	[59]
Salbutamol sulphate sonocrystallized microparticles	Ultrasonication	Isopropyl alcohol	ACI, Rotahaler, gelatin, and 28.3 for 10 sec	44.21 ± 1.30	[60]
Salbutamol sulfate	Mechanofusion Process (dry coating processes)	MgSt	TSI, monodose inhaler device, #3 HPMC, and 60 for 4 sec	75.4 ± 1.00	[61]
Β-Estradiol	SD	Lactose and leucine (6% w/w)	ACI, Spinhaler, #2 gelatin, and 60 TSI, Spinhaler, #2 gelatin, and 60	73.4 ± 1.00 85.0 ± 0.50	[62]

SD: spray drying; co-SD: closed spray drying; SFD: spray freeze drying; LP: lyophilization; PCL: poly(*ε*-caprolactone); CS: cholesterol; DPPC: dipalmitoyl phosphatidylcholine; MgSt: magnesium stearate.

**Table 5:** Microspheres inhaled formulation along with their key findings.

Drug	Method of preparation	Major ingredient	Assembly, device, capsules, and flow rate (L/min)	Percentage of FPF	Reference
*Microspheres*					
Levofloxacin	SD	Chitosan and glutaric acid	NGI, HandiHaler, #3 gelatin, and 60 for 4 sec	32.3 ± 4.0	[65]
Montelukast sodium	SD	Xyloglucan	ACI, Rotahaler, HPMC, and 60 for 4 sec	43.8	[66]
Rifampicin	SE followed by FD	PLGA	NGI and 30 for 10 sec	52.00	[67]
Salbutamol sulfate	RE	Salbutamol sulfate coated by 2% MgSt, polycaprolactone, and PVA	TSI, Rotahaler, #3 gelatin, and 60	15.8 ± 1.5	[68]
Cisplatin	Emulsification and ionotropic gelation method	Chitosan	TSI, Rotahaler, #3 HPMC, and 60	34.4 ± 2.4	[69]

SD: spray drying; FD: freeze drying; RE: rotary evaporation; SE: solvent evaporation; PLGA: poly(lactic-co-glycolic acid); MgSt: magnesium stearate; PVA: poly(vinyl alcohol).

**Table 6:** Summary of the pulmonary delivery of some therapeutic proteins and peptides.

Drug	Method of preparation	Major ingredient	Assembly, device and capsules, flow rate (L/min)	Percentage of FPF	Reference
Glucagon	JM	Pharmatose and erythritol	ACI, Jethaler, #2 HPMC, and 28.3 for 10 sec	33.9	[72]
Glucagon	JM	Citric acid and Respitose® SV003	ACI, Jethaler, #2 HPMC, and 28.3	25.0	[73]
Insulin	SD	Mannitol, bovine serum albumin, and aspartic acid	ACI, Turbospin, #2 HPMC, and 60	83.6 ± 4.7	[74]
Insulin	HPH followed by SD	Phospholipon® 90 H and hydrogenated soy lecithin	MSLI, Aerolizer, #3 HPMC, and 100 for 2.4 sec	63.0 ± 4.0	[75]
Influenza subunit vaccine	SD and SFD	Inulin	MSLI, Twincer®, and 53.7 for 3 sec	37.00 and 23.00	[76]
Pneumococcal surface protein	SD	*S. pneumoniae*	NGI, Cyclohaler, #3 HPMC, and 60 for 4 sec	74.31 ± 1.32	[77]
Unfractionated heparin	co-SD	L-Leucine (1% w/w)	TSI, Aerolizer, #3 gelatin, and 60 for 5 sec	13.23 ± 1.65	[78]
Recombinant human growth hormone	SD	Polysorbate 20, zinc chloride, and lactose	ACI, Spinhaler, #2 gelatin, and 60 for 4 sec	38.52	[79]
Recombinant antigen 85-B	SD	PLGA	ACI and 60 for 4 sec	68.9 ± 8.40	[80]

SD: spray drying; co-SD: closed spray drying; FD: freeze drying; SFD: spray freeze drying; LP: lyophilization; TFE: thin film evaporation; RE: rotary evaporation; SE: solvent evaporation; TFH: thin film hydration; HPH: high pressure homogenizer; CS: cholesterol; SPC: soybean phosphatidylcholine; HPC: hydrogenated phosphatidylcholine; HSPC: hydrogenated soy phosphatidylcholine; DPPC: dipalmitoyl phosphatidylcholine; DPPG: dipalmitoylphosphatidylglycerol.
